# Light harvesting and photoprotective states in the marine diatom *Fragilariopsis* sp.: functional implications of chlorophylls *c*_1_/*c*_2_ in the fucoxanthin–chlorophyll *a*/*c*-binding proteins (FCPs)†

**DOI:** 10.1039/d4ra06711h

**Published:** 2025-02-10

**Authors:** Charalampos Andreou, Constantinos Varotsis

**Affiliations:** a Department of Chemical Engineering, Cyprus University of Technology Limassol Cyprus c.varotsis@cut.ac.cy +357-25002802 +357-25002451

## Abstract

We report pH/pD-dependent fluorescence-excitation spectra of the light-harvesting fucoxanthin–chlorophyll *a*/*c*-binding proteins (FCPs) of the marine diatom *Fragilariopsis* sp. There is a reversible 451 to 455 nm Soret transition accompanied by a 588 to 586 nm Q_x_ transition of chlorophylls *c*_1_/*c*_2_ in the pH/pD 4.9–8 range with a p*K*_a_ = 5.4. The H/D exchangeable site of the 17-acrylate group of chlorophylls *c*_1_/*c*_2_ was characterized and from the pH/pD sensitivity of the Soret and Q_y_ bands we suggest that the chlorophylls *c*_1_/*c*_2_-acrylate-H_2_O moiety can act as a proton acceptor/donor site. Under high intensity light conditions, the acrylate moiety of chlorophylls *c*_1_/*c*_2_ becomes protonated, resembling that observes under acidic conditions. In the photoprotective state, the absorption of chlorophylls *c*_1_/*c*_2_ is red shifted and resembles that observed in the reversible transition from light-harvesting to energy-quenching state at acidic pH. The induced ΔpH and that created from the high intensity light, is responsible for the red-shifted chlorophylls *c*_1_/*c*_2_ due to the protonation of the acrylate group. We present a model that describes an open and a closed form of the protonated/deprotonated chlorophylls *c*_1_/*c*_2_-acrylate-H_2_O moiety that controls the proton loading site in the photoprotective and light harvesting state.

## Abstract 2

Protonic connectivity between R31–Chl *c*_2_ acrylate during pH changes. Two forms (open ↔ closed) of R31–Chl *c*_2_ acrylate ion pair. The closed form has a short distance, *d*, between R31 hydrogen and Chl *c*_2_ acrylate oxygen, and the open form *vice versa*.

## Graphical abstract

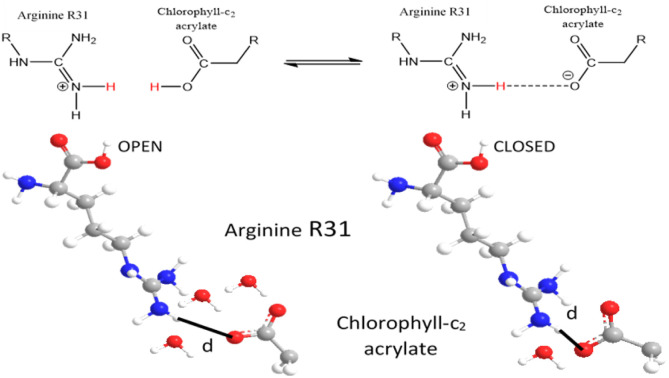

## Introduction

Marine diatoms are involved in major photosynthetic biochemical cycles and carbon fixation.^1^ They perform photosynthesis by utilizing light-harvesting chlorophylls *a*/*c* and fucoxanthins (Fx) located in the plastids and, across their thylakoid membranes, a light induced – proton gradient is formed which is essential for the synthesis of ATP.^1–6^ Marine diatoms contain light-harvesting fucoxanthin–chlorophyll *a*/*c*-binding proteins (FCPs) to collect light energy in the blue-green region that is also available under water, and transfer the trapped energy to the reaction centers where the primary electron transfer reactions convert energy into an electrochemical gradient.^1–6^ In the thylakoid membranes, the pH value of the luminal side decreases upon irradiation until it reaches a steady state under continuous irradiation whereas in the stroma remains unchanged under the same light conditions.^1–3^ The photoacclimation strategy of species growing under variable light conditions enables the efficient regulation of photosystem structures to the amount of absorbed energy.^4–7^ To protect against strong light exposure, diatoms have a nonphotochemical quenching photoprotection system, in which the excess absorbed energy is dissipated as heat.^6,7^ The main NPQ component called energy-dependent quenching (*q*_E_) is triggered by the pH gradient (ΔpH) established between the thylakoid lumen and the stroma during illumination.^8^

The crystal structure of a FCP from the marine *Phaeodactylum tricornutum* revealed the binding of seven chlorophyll *a* (Chls *a*), one chlorophyll *c*_1_ (Chl *c*_1_), one chlorophyll *c*_2_ (Chl *c*_2_), seven fucoxanthin (Fxs) and one diadinoxanthin (Dd) in each monomer subunit.^5^ Efficient energy pathways between Chls *a* and Chls *c* were identified, and each Fx is surrounded by Chls *a*/*c* allowing the energy transfer and quenching *via* Fx.^4–6^ In contrast to the His coordination in Chls *a*/*c* found in the inner antenna of PSII and PSI core, nine Chls *a*/*c* in FCP are coordinated by two His, three Glu, and three Gln residues where Chl *a* 401 is coordinated by a H_2_O molecule.^5^ Hydrophilic groups are present in the tetrapyrrole rings of the two Chls *c*_1_/*c*_2_ which are coordinated by His and Gln and are in close interactions with two Chls *a*. These structural characteristics make Chls *c*_1_/*c*_2_ an efficient harvester of blue-green and even yellow light. Chl *c*_2_ possesses a conjugated to the porphyrin ring at the 8-position vinyl (–CH=CH_2_) group, and Chl *c*_1_ an ethyl group at the same position resulting in a red shifted Chl *c*_2_ Soret band.^5^ The close distances of all Fxs with Chls *a*/*c* indicates that the excess energy absorbed by Chls can dissipated quickly and efficiently through Fxs.^5^ Additional structure–function information related to Chls *c* has been reported, recently.^6^ Specific interactions of the pigments with the protein environment, in addition to pigment–pigment interactions, account for their spectral and redox properties in the FCPs. Among these interactions are the ligands coordinated to Chls *a*/*c* as well as the H-bonding interactions of the pigment groups with the polypeptide side chains.

Specific interactions of the pigment molecules in the protein environment and pigment–pigment interactions account for spectral, and excitation energy transfer efficiency to Chl *a*.^4–9^ The structural differences in Chl *a versus* Chl *c* lead to modified photophysical properties between the different types of macrocycles which have been selected as the active pigments in marine photosynthesis.^4–20^ There is a consensus on the energy transfer pathway that involves Fxs, Chl *c* and Chl *a*.^10–12^ The energy captured by Fx is transferred to Chl *a* on a picosecond time scale and that from Chl *c* to Chl *a* on a femtosecond time scale providing detailed energy flow channels.^10–12,21^ However, there is a conundrum with respect to the time constants of the energy transfer from Fx/Chl *c* to Chl *a* because they are highly dependent on the experimental conditions and the properties of the FCP under investigation.^10–12^ In addition, little information is available related to the dynamics of the pigments and protein residues involved in the transition from light harvesting to photoprotection.^6–14,22,23^

It has been demonstrated that the protonation–deprotonation of 17-acrylate of Chls *c*_1_/*c*_2_ affects their photophysical properties.^17^ There are two marker transitions to characterize the dynamics of Chls *c*_1_/*c*_2_ in the FCPs of diatoms. The first is related to the protonation/deprotonation of the acrylate group causing a red shift in the Soret and Q_x_ bands and the second is the red-shift in the Soret and Q_x_/Q_y_ bands of Chl *c*_2_ with respect to Chl *c*_1_.^17,18,24^ The pH 4.9 minus pH 8 fluorescence-excitation difference spectra observed at 638 nm showed a broad peak around 471 nm representing a mixture of protonated and deprotonated 17-CH=CH–COO^−^ at a ratio of 3 : 1 and also a negative peak at 446 nm representing a deprotonated 17-CH=CH–COO^−^.^17^ On the same line, quantum mechanical/molecular mechanical calculations have shown that the absorption wavelength of the Soret band of protonated Chl *c* is 25 nm longer than that of deprotonated Chl *c*, which is due to the delocalization of the lowest (LUMO) and second lowest (LUMO+1) unoccupied molecular orbitals toward the acrylate group.^18^ It was suggested that in the FCP, the decrease in pH on the luminal side under high-intensity light (HL) conditions leads to protonation of the acrylate moiety and thereby a red shift in the absorption wavelength. In addition, it was reported that the energy transfer efficiency from Chl *c* to Chl *a* is reduced under acidic conditions. It is therefore of pivotal importance to have full characterization of the structure–function relationship of the Chls *c* in the FCP complex including the properties of the acrylate moiety.

In the work presented here, we have extended our work and employed fluorescence spectroscopy to probe the pH/pD-dependent fluorescence-excitation spectra of the light-harvesting fucoxanthin–chlorophyll *a*/*c*-binding proteins (FCPs) of the marine diatom *Fragilariopsis* sp.^25,26^ There is a reversible Soret transition from 451 to 455 nm in the pH/pD 4.9–8 range accompanied by a pH/pD sensitive Q_y_ transition from 588 to 586 nm of Chls *c*_1_/*c*_2_ with a p*K*_a_ = 5.4. The pH/pD dependent fluorescence-excitation spectra, revealed H/D exchangeable proton sites in the moiety of the 17-acrylate group of Chls *c*_1_/*c*_2_. In addition, the acidic pH created under HL conditions is also involved in the protonation of the 17-acrylate group of Chl *c*_1_/*c*_2_ that creates the red-shift in the absorption spectrum. We propose a mechanism in which the exchangeable proton sites in the acrylate moiety of Chl *c*_1_-K136 and Chl *c*_2_-R31 exist in a closed and an opened form. The ion pairs can exist in a fully protonated opened form and/or in a closed form in which the protonated forms of lysine K136 H^+^ and arginine R31 H^+^ interact with the deprotonated acrylate moieties of Chl *c*_1_ and Chl *c*_2_, respectively.

## Materials and methods

The culture of *Fragilariopsis* sp. (CCAP 1029/24) was obtained from CCAP (Culture Collection of Algae and Protozoa). Cells were grown in f/2-Si medium, at 19 °C in a dark–light cycle (12 h:12 h) under white LED light.^27^ The light intensity used for the growth of the cells culture was 150 μmol photons per m^2^ per s for the low-intensity light (LL) condition and 350 μmol photons per m^2^ per s for the high-intensity light (HL). Cells from *Fragilariopsis* sp. diatom were harvested by centrifugation (8000 rpm, 30 min, 4 °C) and the supernatant medium was removed. The cells were resuspended in 20 mM Tris and sonicated in an ice bath for 20 minutes. Thylakoid membranes were then solubilized with the addition of β-1,4-dodecyl maltoside (β-DDM, 30 mg, 4 mM) in the resuspended cells while they were shaken for 20 minutes on ice.^28^ Separation of solubilized FCPs was carried out on an ion exchange column (HiPrep Q HP 16/10, 20 mL) in 25 mM Tris, 2 mM KCl and 0.4 mM β-DDM at pH 7.4 using a Shimadzu LC 20AD with a SPD-20A detector with two-wavelength detection. The samples were loaded in a column, and fractions were eluted using a gradient from 0 to 750 mM KCl in a buffer made of 25 mM Tris, 750 mM KCl and 0.4 mM β-DDM, pH 7.4 at a flow rate of 3 mL min^−1^. Fractions were pooled and concentrated using Amicon filtration devices with a cutoff of 10 kDa and characterized, spectroscopically. The rest of the fractions were stored at −80 °C until further use.

A series of buffer solutions with different pH values were prepared using 25 mM of MES-NaOH (pH 4.9–6.4) or Tris–HCL (pH 8) at room temperature. Absorption spectra were recorded at room temperature with a Cary 60 UV-vis spectrometer (Agilent Technologies, USA). The buffer prepared in D_2_O was measured assuming pD = pH (observed) + 0.4. For hydrogen/deuterium studies, solutions of the FCP were exchanged three times in 99.9% D_2_O. Fluorescence and fluorescence-excitation spectra were measured in a 3 mm cuvette, at room temperature, with a Cary Eclipse Fluorescence Spectrophotometer (Agilent Technologies, USA). The excitation and emission bandwidth slits were 10.0 nm and 5.0 nm, respectively.

## Results

**Fig. 1:**
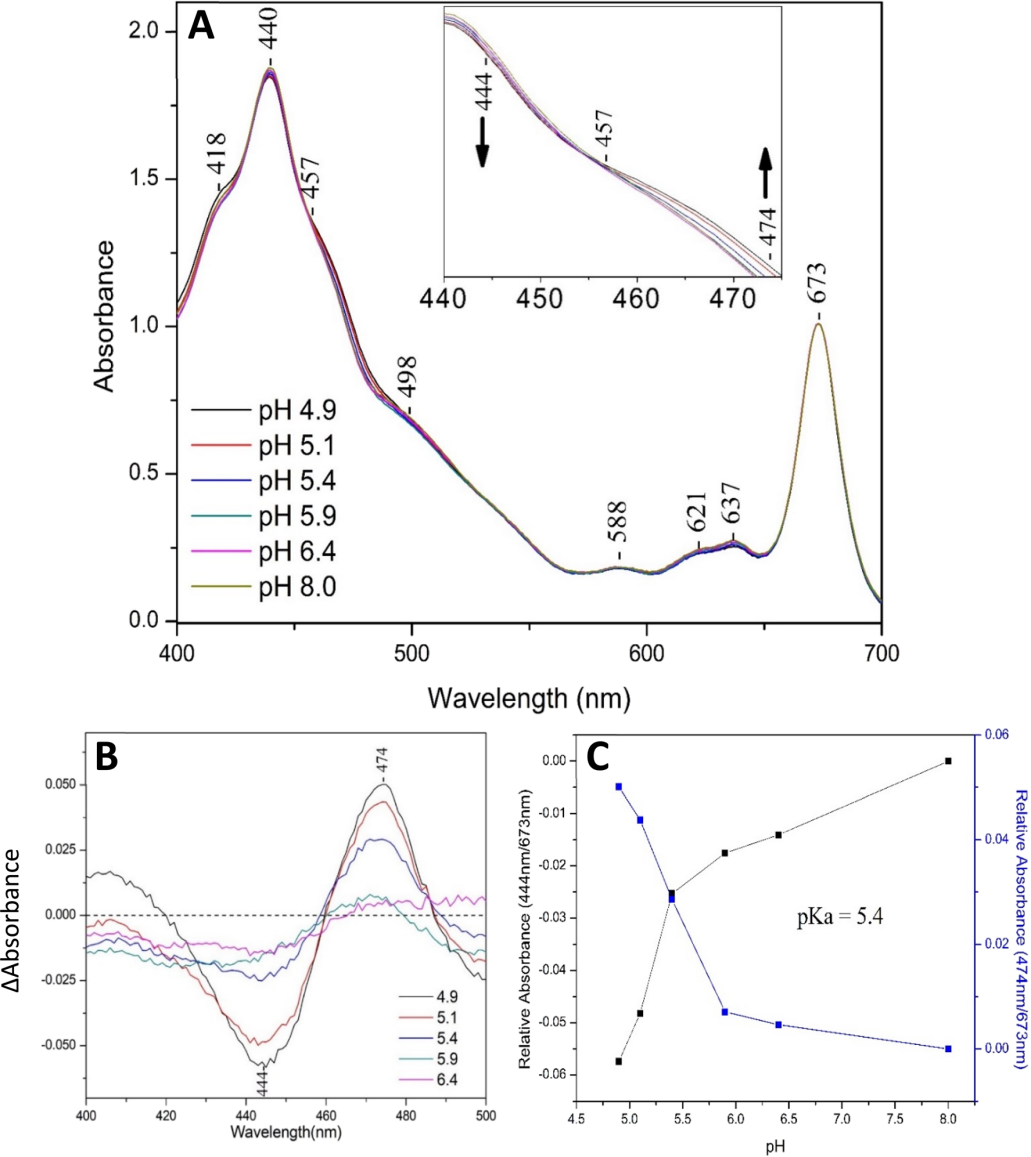
Panel (A): absorption spectra of FCPs of *Fragilariopsis* sp. normalized at 673 nm. The inset shows the enlarged spectra highlighting the isosbestic point. Panel (B): difference absorption spectra obtained by subtracting the spectra measured at pH 8 from those with pH values designated in the figure. Panel (C): absorbance at 444 nm (black squares) and 474 nm (blue squares) relative to that at 672 nm against the pH values. The p*K*_a_ value was determined from the graph.

Fig. 1, panel A, shows the UV-vis spectra of a fraction of solubilized FCPs of *Fragilariopsis* sp. at room temperature in the pH range 4.9–8. The transitions at 440 (Soret), 621 (Q_x_) and 673 (Q_y_) nm accompanying pH changes are attributed to Chl *a*, and at 457 (Soret), 588 (Q_x_) and 637(Q_y_) nm to Chls *c*_1_/*c*_2_.^8,17,25,26^ The broad transition in the 500–560 nm range has been attributed to red Fxs, as blue Fxs absorb in the 420–470 nm region.^8,25^ The inset depicts the pH dependent behavior of the Soret band as the pH is decreased from 8 to 4.9 with isosbestic point at 457 nm. Panel B shows the difference spectra obtained from the subtraction of the pH 8 spectrum from the respective spectra in the pH order 4.9, 5.1, 5.4, 5.9, 6.4. A peak/trough at 474/444 nm is formed as the pH decreases from 8 to 4.9. In ESI Fig. S1† the same results were obtained when the absorbance at 672 nm kept below 1. The data demonstrate the transformation of one species to another in a first order equilibrium, as a change of pH. We attribute the observed changes, in agreement with previous work,^17^ to protonation (17-CH=CH–COOH) and deprotonation (17-CH=CH–COO^−^) of the acrylate moiety of Chls *c*_1_/*c*_2_ in the FCP. In panel C, the absorbances of the FCP at 444 (black line) and at 474 (blue line) were plotted against the pH in the 4.9 to 8 range and a p*K*_a_ = 5.4 was determined. The changes in the absorption of 444 and 474 nm are attributed to the acid-dissociation of Chl *c*_1_/*c*_2_. Obviously, the 17-acrylate group of Chls *c*_1_/*c*_2_, as it is in the case of the FCP from *C. calcitrans*, controls the acidic and basic forms of Chls *c*_1_/*c*_2_ and from the analysis of the fluorescence data presented below, reversibly.^17^

### Determination of the relative concentration of protonated Chls *c*

In an acid-dissociation equilibrium, the Henderson–Hasselbalch equation defines pH using the p*K*_a_ and the concentrations of both the starting acid and the conjugated base: pH = p*K*_a_ + log([RCOO]/[RCOOH]), where pH is the negative logarithm of [H^+^], p*K*_a_ is the acid dissociation constant, and [RCOO] and [RCOOH] are the concentrations of the conjugate base and acid, respectively. Applying the relation of [RCOO] + [RCOOH] = *N*, where *N* is a constant value, the concentration of the starting acid is written as:[RCOOH] = *N*/{1 + 10^pH−p*K*_a_^}.[RCOOH] = 100/{1 + 10^4.9−5.4^}[RCOOH] = 75.97 ≈ 76%

By using the determined p*K*_a_ value of 5.4 from Fig. 1, panel C, the relative concentration of protonated Chls *c*_1_/*c*_2_ (17-CH=CHCOOH) was calculated to be 76% and 0% at pH 4.9 and 8, respectively. Consequently, the fluorescence excitation spectrum at pH 8 represents absorbance of the deprotonated form of Chl *c*_1_/*c*_2_ (17-CH=CH–COO–) and that at pH 5 consists of absorbances from protonated and deprotonated Chls *c*_1_/*c*_2_ at a ratio of 3 : 1.

**Fig. 2:**
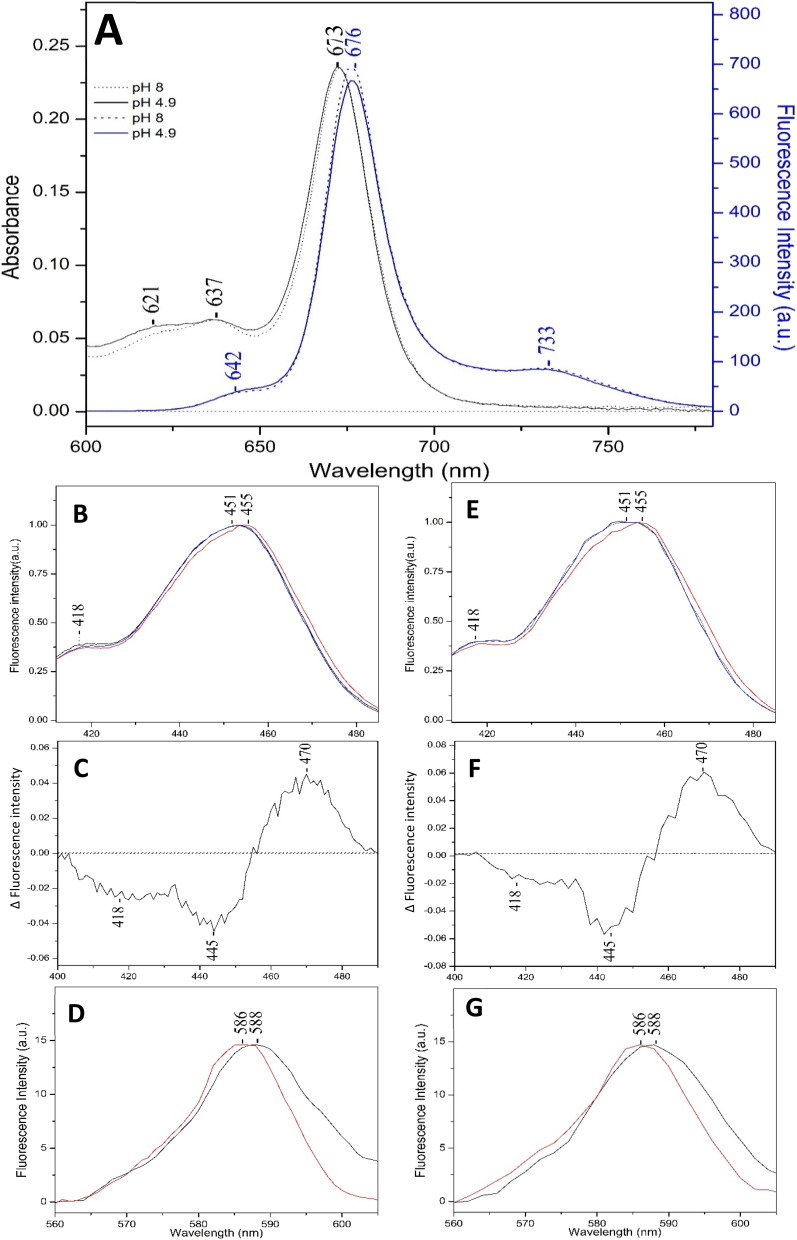
Panel (A): absorption (black line) and fluorescence spectra (blue line) of FCPs of *Fragilariopsis* sp. at pH 8 (broken line) and 4.9 (solid line) excited at 457 nm. Panel (B): fluorescence excitation spectra from the spontaneous emission from Chl-*c*_1_/*c*_2_ at 640 nm at pH 8 (black line) and pH 4.9 (red line). The blue line spectrum is from a sample that was original at pH 8 and subsequently was reduced to 4.9 reproducing the original (red line) spectrum, and finally re-adjusted back to pH 8 reproducing the original (black line) spectrum obtained at pH 8. The spectra were normalized at maxima. (C) Difference pH 4.9 minus pH 8 spectrum. (D). Emission at 640 nm of Q_x_ of Chl-*c*_1_/*c*_2_ at pH 4.9 (black line) and pH 8 (red line). (E) Fluorescence excitation spectra from the spontaneous emission from Chl *c*_1_/*c*_2_ at 640 nm at pD 8 (black line) and pD 4.9 (red line). The blue line spectrum is from a sample that was original at pD 8 and subsequently was reduced to pD 4.9 reproducing the original (red line) spectrum, and finally re-adjusted back to pD 8 reproducing the original (black line) spectrum obtained at pD 8. The spectra were normalized at maxima. (F) Difference pD 4.9 minus pD 8 spectrum. (G) Emission at 640 nm of Q_x_ of Chl-*c*_1_/*c*_2_ at pD 4.9 (black line) and pD 8 (red line).

Fig. 2, panel A, shows the fluorescence spectra of FCP at pH 4.9 (blue solid line) and at pH 8 (blue broken line) excited at 457 nm and the absorption spectra at pH 4.9 (black solid line) and at pH 8 (black broken line). Both fluorescence spectra show a peak at 676 nm that originate from the Q_y_ transition of Chl *a* bound to FCP and a broad band at 733 nm in agreement with those reported for *C. gracilis* and *C. calcitrans*.^17,21^ In addition, there is a broad peak at 642 nm indicating the spontaneous emission from the Q_y_ transition of Chls *c*_1_/*c*_2_, which is also in agreement with previous results.^17–21^ The Q_y_ absorption of Chl *a* at 672 nm is identical at pH 4.9 and pH 8. In panel B, the fluorescence excitation spectra from the spontaneous emission from Chl *c*_2_ at 640 nm at pH 8 (black line) and pH 4.9 (red line) are presented. The blue line spectrum is from a sample that was original at pH 8 and subsequently was reduced to 4.9 reproducing the original (red line) spectrum, and finally re-adjusted back to pH 8 reproducing the original (black line) spectrum obtained at pH 8. This observation demonstrates that the pH 8 to pH 4.9 transitions are reversible. The spectra were normalized at maxima. Three distinct transitions at 451, 455 and 418 nm are observed. The spectral shape of the spectra in panel B are well matched with those obtained in ref. 17 and demonstrate that originate from the Soret transitions of Chls *c*_1_/*c*_2_ bound to FCP. Therefore, the spectra observed at 640 represent the absorption spectra of Chls *c*_1_/*c*_2_. In the spectrum at pH 4.9, the weak peak at 418 nm and that at 451 nm have lost intensity and there is a 3–4 nm red-shifted inhomogeneous broadening of the peak centered at 455 nm. We assign the reversible 451 to 455 nm pH-dependent transition of Chls *c*_1_/*c*_2_ to the deprotonation/protonation of the 17-acrylate group, in agreement with previous work on *C. calcitrans*.^17^ The blue-shifted pH-sensitive transition at 418 nm is also observed and it was also present in the fluorescence excitation spectra of *C. calcitrans* but not reported in ref. 17. The pH 4.9 and pH 8 spectra were normalized at maxima. The pH 4.9 minus pH 8 spectrum presented in panel C shows a positive band at 470 nm and negative bands at 445 and 418 nm. The origin of the blue shifted Chls *c*_1_/*c*_2_ at 418 nm is not certain. Modifications of the peripheral groups, the coordination, ligand binding to Mg and H-bonding interactions with the protein environment can cause the observed shift at 418 nm. In panel D, the fluorescence excitation spectra at 640 nm of Q_x_ of Chls *c*_1_/*c*_2_ at pH 4.9 (black line) and pH 8 (red line) are presented and further support the pH sensitivity of Chls *c*_1_/*c*_2_. In panel E, the fluorescence excitation spectra from the spontaneous emission from Chls *c*_1_/*c*_2_ at 640 nm at pD 8 (black line) and pD 4.9 (red line) are presented. The blue line spectrum is from a sample at pD 8 that it was subsequently reduced to pD 4.9 reproducing the original (red line) spectrum, and finally it was re-adjusted back to pD 8 reproducing the original (black line) spectrum obtained at pD 8. This observation demonstrates that the pD 8 to pD 4.9 transitions are reversible in agreement with the pH experiments presented in panel B. The spectra were normalized at maxima. It should be noted that the sample at pD 4.9 was made from a sample of pH 8, in which the acrylate is deprotonated and subsequently it was reduced to pD 4.9. This implies that the observed differences are due to the deuterated acrylate group of Chl *c*_2_. Panel F shows the difference pD 4.9 minus pD 8 spectrum, and finally panel G depicts the emission at 640 nm of Q_y_ of Chls *c*_1_/*c*_2_ at pD 4.9 (black line) and pD 8 (red line).

**Fig. 3:**
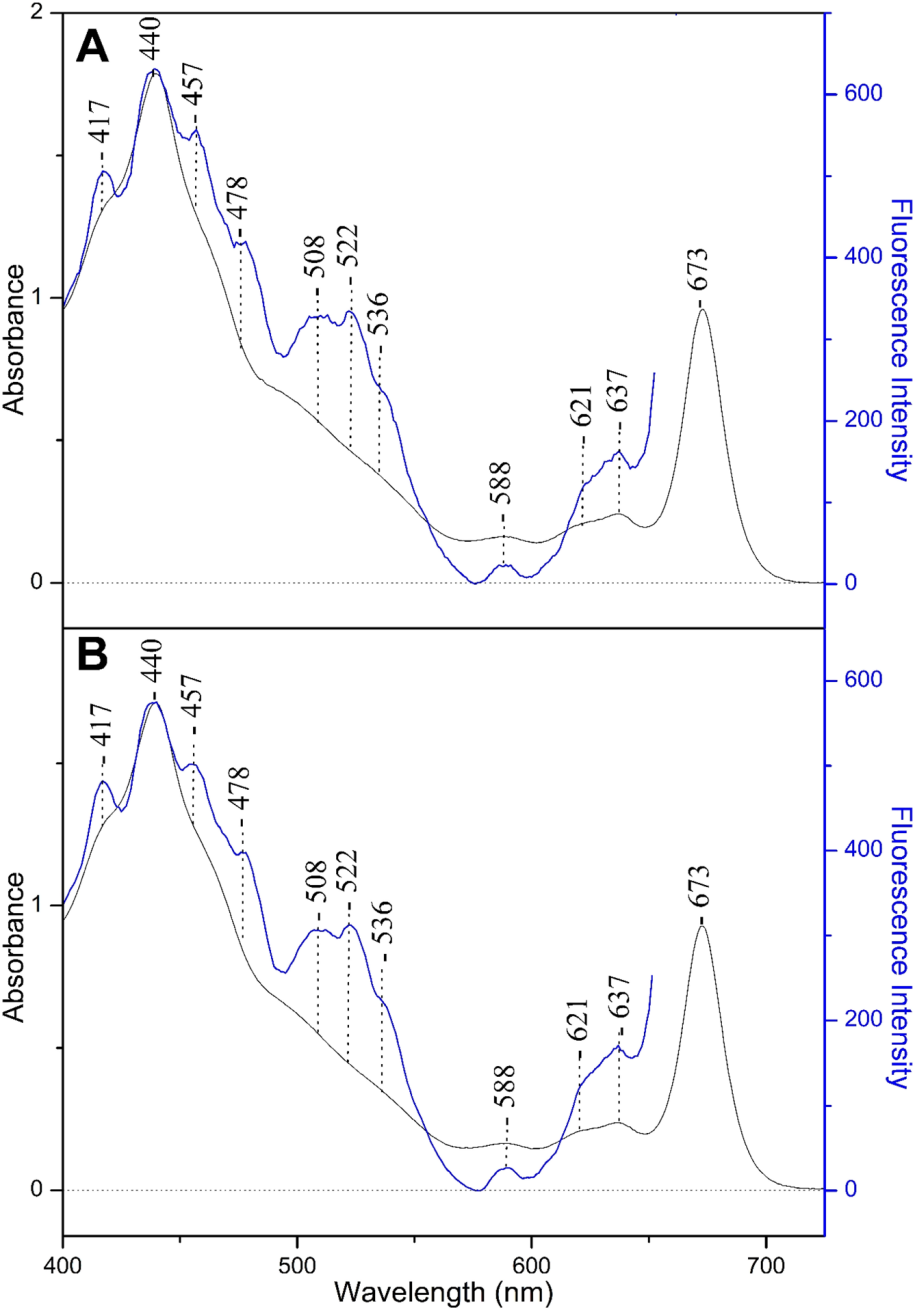
Panel (A): fluorescence excitation from the spontaneous emission at 677 nm (blue line) and absorbance spectrum (black line) of a fraction of FCPs of *Fragilariopsis* sp. at pH 8. Panel (B): fluorescence excitation from the spontaneous emission at 677 nm (blue line) and absorbance spectrum (black line) at pH 4.9. The spectra were normalized at the maxima.

Fig. 3 shows the fluorescence excitation spectra at 677 nm (blue line) from a fraction of solubilized FCP of *Fragilariopsis* sp., and for comparison the absorption spectrum (black line) at pH 8 (panel A) and pH 4.9 (panel B). The fluorescence excitation spectra at 677 nm show that there is good agreement with the absorptivity, and sufficient energy transfer to Chls *a* takes place when Chls *c*_1_/*c*_2_ and Fxs were excited. In particular, the 508, 522 and 536 nm peaks are due to the lower energy red Fxs exhibiting different interactions with nearby residues whereas that at 478 nm has contributions from both Fx and Chls *c*_1_/*c*_2_. These observations are in good agreement with those previously reported UV-vis and fluorescence excitation spectra for FCPs from different diatoms grown under LL conditions where it was shown that there is a strong coupling between Fxs and Chls *a*, as well as, between Chls *c*_1_/*c*_2_ and Chls *a*.^25^ In panel B, the pH 4.9 fluorescence excitation spectra at 680 nm and the absorption spectra are similar to those at pH 8 presented in panel A. Similar results were obtained when the absorbance at 672 nm kept below 1 with and without normalization (ESI Fig. S2/S3†).

**Fig. 4:**
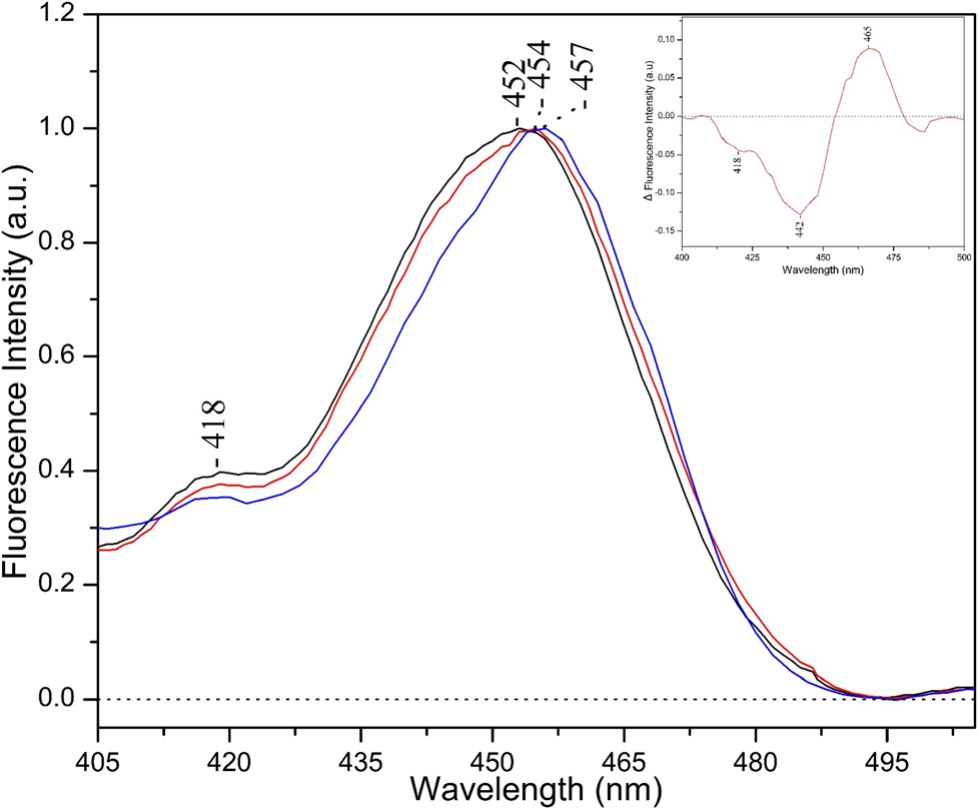
Fluorescence excitation spectra from the spontaneous emission from Chl-*c*1/*c*2 at 640 nm of *Fragilariopsis* sp. FCP at low intensity light pH8 (black), pH 4.9 (red) and high intensity light pH8 (blue) normalized at maxima. The inset shows the difference of the emission spectra 640 nm between the high intensity light at pH8 minus the low intensity light at pH 8.

Fig. 4 compares the emission at 640 nm of the FCP of *Fragilariopsis* sp. at pH 8 (black line) and at pH 5 (red line) under LL growth condition with that observed under HL growth condition (blue line). The inset depicts the pH 8 species grown under high intensity light minus the pH 8 species grown under low intensity light. The comparison of the difference spectra between the inset in Fig. 4 and the Fig. 2C demonstrates, that under high intensity light conditions, the absorption of chlorophylls *c*_1_/*c*_2_ is red shifted and resembles that observed in the reversible transition from light-harvesting to energy-quenching state at acidic pH in which the acrylate moiety of chlorophylls *c*_1_/*c*_2_ is protonated, resembling that observed under acidic conditions.

## Discussion

The observation of three Soret absorption bands of Chls *c*_1_/*c*_2_ in the protein environment of *Fragilariopsis* sp. and their similarities with those observed in *C. calcitrans* gives a new insight into the photosynthetic properties of the bound Chls *c*_1_/*c*_2_ to FCPs. Of particular note is the observation that at pH 4.9 there is an increase in the fluorescence intensity at 642 nm resembling that observed in cells from *Thalassiosira pseudonana* grown under HL.^25^ Therefore, Chls *c*_1_/*c*_2_ in the isolated FCPs and those embedded in the thylakoid membranes have similar interactions with the protein environment leading to the observed decrease in energy transfer efficiency to Chl *a*. On this line, it is well known that in the thylakoid membranes, the pH value of the luminal side decreases upon irradiation.^1–6,18^ The peaks/troughs shown in Fig. 3, panels C and F, are not only unaffected by the pH/pD exchanges in the 4.9–8 range but also demonstrate that all the processes are reversible indicating that the acrylate moiety is easily accessible to H_2_O/D_2_O exchanges. A sequential or concerted H-bonded connectivity between the environments sensed by the Chl *c*_2_-acrylate-R31-H_2_O and Chl *c*_1_-acrylate-K136 could have an activation energy for proton motion that leads to the identification of a proton channel and H_2_O molecules. The presence of H_2_O/D_2_O molecules in the Chls *c*_1_/*c*_2_ acrylate moieties in addition of being able to transfer a proton across a prearranged water array can also induce Chls *c*_1_/*c*_2_-linked ionization phenomena. We suggest that a change in the acrylate environments can be communicated to both the distal and proximal environment of Chls *c*_1_/*c*_2_, causing a modification of the H-bonding status of the proximal and distal coordinated ligands (His 39) to Mg of Chl *c*_2_ and Cln143 to Mg of Chl *c*_1_. This way, the optical transition will shift to higher energy as a result of the increased splitting of the d orbitals and the energy of the antibonding π* orbitals. The data for heme-copper oxidases and for the peroxidase class of enzymes show that such heme-linked ionization phenomena exist.^29^

### Exchangeable protons

**Fig. 5:**
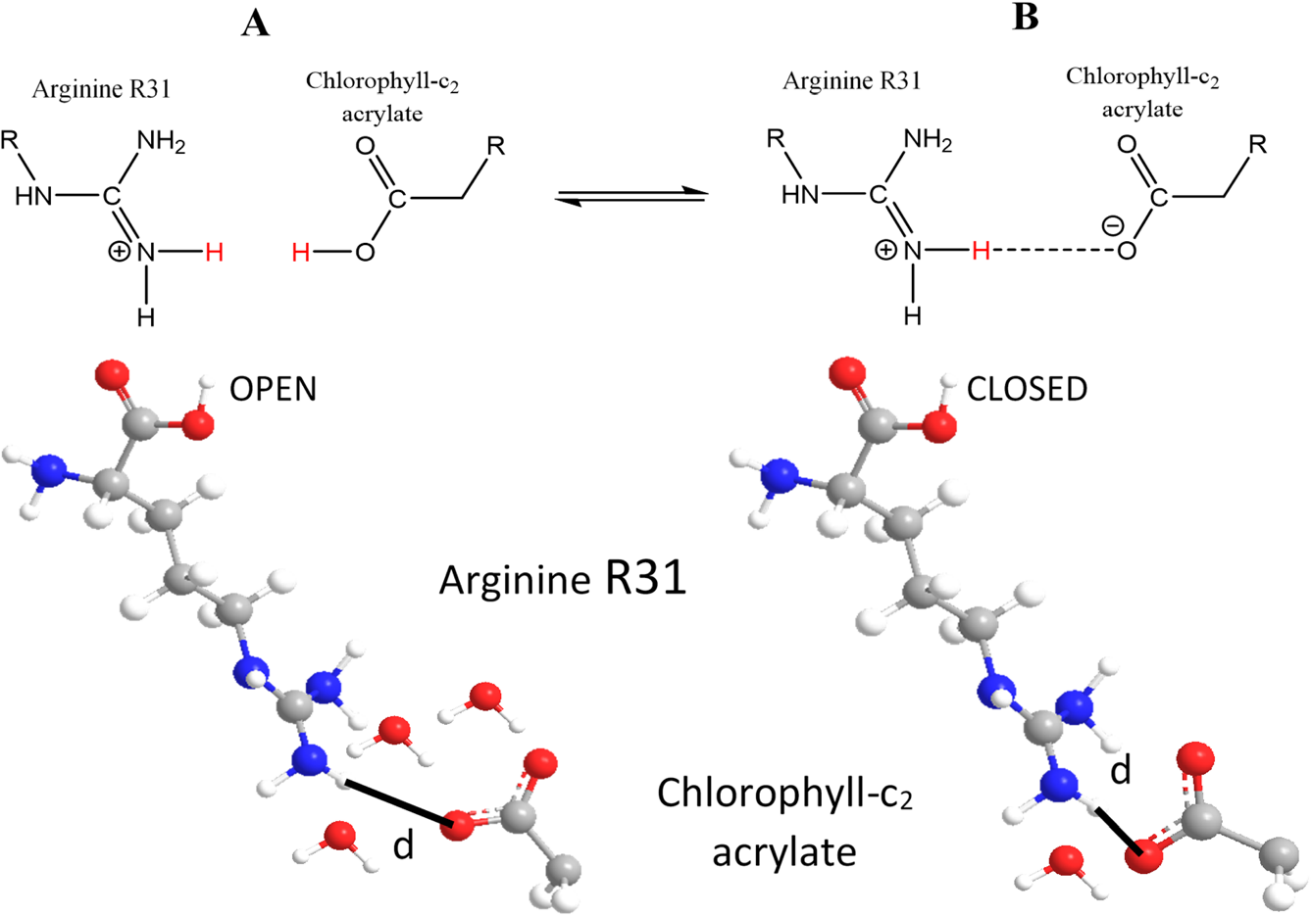
Schematic view of the protonic connectivity between the arginine R31 and chlorophyll *c*_2_ acrylate moiety during pH changes. Two forms (open and closed) of the R31/Chl-*c*2 acrylate ion pair. The closed form is on the right with a short distance, marked d, between the 2HH2 hydrogen of the arginine and the O2H oxygen of the chlorophyll-*c*_2_ acrylate. The open gate form is shown on the left.

The QM/MM-optimized structures of deprotonated acrylate Chls *c*_1_/*c*_2_ showed similar distances (2.8 Å) between the acrylate and arginine R31 (Chl *c*_2_) and Lysine K136 (Chl *c*_1_).^18^ In the protonated forms of Chls *c*_1_/*c*_2_ the distance between acrylate and R31 (Chl *c*_2_) is 7.7 Å whereas that of acrylate and K136 (Chl *c*_1_) is 4.2 Å. In Fig. 5 we report a model in which for simplicity we show only the protonated and deprotonated forms of Chl *c*_2_ that interact with H_2_O molecules and R31 forming an open and a closed gate, forms. The corresponding Chl *c*_1_-H_2_O-K136 interactions are not shown. Water molecules between redox chromophores can affect the electron transfer as well as the triplet–triplet energy transfer. The interaction of intervening H_2_O molecules, as well undetectable by X-ray crystallography mobile H_2_O molecules, located in hydrophobic cavities can play a crucial role in transferring protons from amino acid residues involving a redox state-dependent switch in the orientation of these water molecules and/or forming H-bonding networks with other protein residues. The close proximity of the R31 residue with the acrylate moiety provides a gate for H^+^/H_2_O motion, and suggests that they form an ion pair. Dissociation of the pair is expected to raise the p*K*_a_ of the acrylate substantially, and lower the p*K*_a_ of R31. We propose that the pH sensitivity has an effect on the p*K*_a_ of specific groups resulting in changes in the protonation pattern, which in turn affects local structure *via* changes in hydrogen-bonding pattern and H_2_O motion that affects the energy transfer under acidic conditions. The protonation/deprotonation events in the 17 acrylate-R31/H_2_O site are fundamental to the function of diatoms because pH and water molecules play an important role in the water transfer that affects the triplet–triplet energy transfer properties and thus, the switch of the FCP from light-harvesting to energy-quenching state. The abovementioned analysis is also suggested to occur in the vicinity of Chl *c*_1_-H_2_O-K136 but to a lesser extend due to the shorter 17-acrylate-K136 distance (4.2 Å) in the protonated form (see below).

Based on QM/MM optimized structures of FCP the optimized geometry showed that salt bridges form between the acrylate moiety of Chl *c*_2_ and R31 and, Chl *c*_1_ and K136 when Chl *c*_1_ and Chl *c*_2_ are deprotonated, respectively.^18^ In addition, it was reported that an increase in the calculated absorption wavelength upon the protonation of Chls *c*_1_/*c*_2_ is also observed in the absence of the FCP environment. Thus, the absorption wavelength shift of Chls *c*_1_/*c*_2_ predominantly originates from the change in the protonation state of the acrylate moiety instead of the protein environment. Of note, is the calculated difference in the increase of the absorption wavelength between the protonated and deprotonated forms of Chl *c*_1_ (+31 nm) and Chl *c*_2_ (+18 nm). Our data suggest that the protonated/deuterated form is not influenced by the pH/pD exchanges in the 4.9–8 range. We suggest that the presence of H_2_O/D_2_O molecules in the moiety of R31-Chl *c*_2_- and K136-Chl *c*_1_ acrylate controls the deprotonated (*i.e.* salt bridge) to protonated transition of acrylate (*i.e.* in the absence of salt bridge) is highly dependent of the protein environments. On this line, the dissociation of the ion pair can create a transient proton loading site. The optimized QM/MM structures of the FCP showed an increase in the R31 to 17-acrylate distance from 2.8 Å in the deprotonated form to 7.7 Å in the protonated form whereas the corresponding Chl *c*_1_-acrylate to K136 increased from 2.7 to 4.2 Å, only.^18^ This suggests that the protein environment of Chl *c*_1_ is more difficult to protonate/deuterate the acrylate moiety than the corresponding protein environment of Chl *c*_2_. In this scenario, we suggest that the acrylate of Chl *c*_2_ 403 in conjunction with H_2_O molecules and/or proton channels is the predominant proton loading site providing a protonic switch that is part of the regulation from the light harvesting to the photoprotective states. The data suggest that at low pH there is an overload of H_2_O molecules, as compared to high pH, that control the open and closed forms. This emphasizes the importance of balance in the dynamics of water presence in the R31-acrylate moiety to retain the gate in the open form, photoprotective mode. Chlorophylls *c* have been identified in both the stromal side and in the luminal side of PSII-FCP.^22,23^ Furthermore, the release of protons to the luminal side of the thylakoid membrane in the chloroplasts results in lowering the pH and depends on the light intensity. Under low light conditions Chls *c*_1_/*c*_2_ are deprotonated whereas under high light conditions the pH is decreased and becomes protonated. Although not included in the model, the nearby Fx306 residue interacts through H-bonding with Chl *c*_2_.^18^ This way, the role of Fx 304 is to fix Chl *c*_2_ in a certain configuration and distance from R31. This way, the pH induced changes between the deprotonated (closed form) and protonated (open form) Chl *c*_2_ 403 at the acrylate moiety controls the Chl *c*_2_-R31 distance. There is consensus that the pH-triggered activation of the rapidly inducible thermal dissipation of excess absorbed energy (*q*_E_) should be conserved in diatoms.^30^ We suggest that the H-bonding of the acrylate moiety of Chls-*c*_1_/*c*_2_ to the red Fxs 307/306 residues could play an important role in proton motion in a water pool, as it has been demonstrated in the oxidative or reductive phases in cytochrome oxidase that contains H_2_O channels and maintain a transmembrane proton gradient (ΔpH).^31–34^ We suggest that Chl *c*_2_ and more specifically the 17-acrylate moiety has a role in the photoprotection in diatoms, which switches the FCP function between light harvesting and energy-dissipation modes depending on the light intensity. We propose that H_2_O molecules are involved in controlling the extend of the photoprotection mode which is characterized as the open protonated form with a larger distance between R31 and 17-acrylate Chl*c*_2_ 403 as compared to the deprotonated closed form.

## Data availability

The authors Charalampos Andreou and Constantinos Varotsis confirm that the data supporting the findings of the study with title ‘Light harvesting and photoprotective states in the marine diatom *Fragilariopsis* sp.: functional implications of chlorophylls *c*_1_/*c*_2_ in the fucoxanthin–chlorophyll *a*/*c*-binding proteins (FCPs)’ are available within the article.

## Author contributions

CA performed the experiments and analyzed the results and CV analyzed the results and wrote the paper.

## Conflicts of interest

The authors declare that the research was conducted in the absence of any commercial or financial relationships that could be construed as a potential conflict of interest.

## Supplementary Material

RA-015-D4RA06711H-s001

## References

[cit1] Büchel C. (2019). Science.

[cit2] Büchel C. (2020). Adv. Photosynth. Respir..

[cit3] Büchel C. (2020). Biochim. Biophys. Acta, Bioenerg..

[cit4] Pi X., Zhao S., Wang W., Liu D., Xu C., Han G., Kuang T., Sui S., Shen J. R. (2019). Science.

[cit5] Wang W., Yu L.-J., Xu C., Tomizaki T., Zhao S., Umena Y., Chen X., Qin X., Suga M. (2019). *et al.*. Science.

[cit6] Feng Y., Li Z., Li X., Shen L., Liu X., Zhou C., Zhang J., Sang M., Han G., Yang W. (2023). *et al.*. Sci. Adv..

[cit7] Nagao R., Ueno Y., Yokono M., Shen J. R., Akimoto S. (2018). Biochim. Biophys. Acta, Bioenerg..

[cit8] Buck J. M., Kroth P. G., Lepetit B. (2021). Plant J..

[cit9] Wang W., Zhao S., Pi X., Kuang T., Sui S. F., Shen J. R. (2020). FEBS J..

[cit10] Gelzinis A., Butkus V., Songaila E., Augulis R., Gall A., Buchel C., Robert B., Abramavicius D., Zigmantas D., Valkunas L. (2015). Biochim. Biophys. Acta, Bioenerg..

[cit11] Nagao R., Ueno Y., Yokono M., Shen J. R., Akimoto S. (2019). Photosynth. Res..

[cit12] Papagiannakis E., van Stokkum I. H. M., Fey H., Büchel C., van Grondelle R. (2005). Photosynth. Res..

[cit13] Oka K., Ueno Y., Yokono M., Shen J. R., Nagao R., Akimoto S. (2020). Photosynth. Res..

[cit14] Chrysafoudi A., Maity S., Kleinekathöfer U., Daskalakis V. (2021). J. Phys. Chem. Lett..

[cit15] Ikeda Y., Yamagishi A., Komura M., Suzuki T., Dohmae N., Shibata Y., Itoh S., Koike H., Satoh K. (2013). Biochim. Biophys. Acta, Bioenerg..

[cit16] Nagao R., Yokono M., Ueno Y., Shen J.-R., Akimoto S. (2019). J. Phys. Chem. B.

[cit17] Yamano N., Mizoguchi T., Fujii R. (2018). J. Photochem. Photobiol., A.

[cit18] Tsujimura M., Sugano M., Ishikita H., Saito K. (2023). J. Phys. Chem. B.

[cit19] Nagao R., Yokono M., Tomo T., Akimoto S. (2014). J. Phys. Chem. Lett..

[cit20] Nagao R., Yokono M., Ueno Y., Shen J. R., Akimoto S. (2020). J. Phys. Chem. B.

[cit21] Nagao R., Yokono M., Ueno Y., Akimoto S., Tomo T. (2013). J. Phys. Chem. B.

[cit22] Nagao R., Kato K., Kumazawa M., Ifuku K., Yokono M., Suzuki T., Dohmae N., Akita F., Akimoto S., Miyazaki N. (2022). *et al.*. Nat. Commun..

[cit23] Nagao R., Kato K., Ifuku K., Suzuki T., Kumazawa M., Uchiyama I., Kashino Y., Dohmae N., Akimoto S., Shen J. R. (2020). *et al.*. Nat. Commun..

[cit24] Faqley M. W. (1988). Plant Physiol..

[cit25] Tselios C., Varotsis C. (2022). RSC Adv..

[cit26] Andreou C., Tselios C., Ioannou A., Varotsis C. (2023). J. Phys. Chem. B.

[cit27] Guillard R. R. , Culture of Phytoplankton for Feeding Marine Invertebrates, in Culture of Marine Invertebrate Animals, ed. W. L. Smith and M. H. Chanley, Springer, Boston MA, 1975, pp. 29–60

[cit28] Beer A A., Gundermann K., Beckmann J., Büchel C. (2006). Biochemistry.

[cit29] Pinakoulaki E., Pfitzner U., Ludwig B., Varotsis C. (2023). J. Biol. Chem..

[cit30] Buck J. M., Sherman J., Río Bártulos C., Serif M., Halder M., Hankel J., Falciatore A., Lavaud J., Gorbunov M., Kroth P. (2019). *et al.*. Nat. Commun..

[cit31] Schelvis H., Varotsis C., Deinum G., Ferguson-Miller S., Babcock G. (1997). J. Am. Chem. Soc..

[cit32] Stavrakis S., Pinakoulaki E., Urbani A., Varotsis C. (2002). J. Phys. Chem. B.

[cit33] Koutsoupakis C., Soulimane T., Varotsis C. (2003). J. Biol. Chem..

[cit34] Vamvouka M., Varotsis C. (1999). J. Phys. Chem..

