# Supplementary material for: Light harvesting and photoprotective states in the marine diatom Fragilariopsis sp.: functional implications of chlorophylls c1/c2 in the fucoxanthin–chlorophyll a/c-binding proteins (FCPs)
Source: RSC Adv. 2025 Feb 10;15(6):4322–30. doi: 10.1039/d4ra06711h (PMC11808296; doi:10.1039/d4ra06711h)
Supplement: RA-015-D4RA06711H-s001 [file RA-015-D4RA06711H-s001.pdf]

Supplementary Information (SI) for RSC Advances.
This journal is © The Royal Society of Chemistry 2025

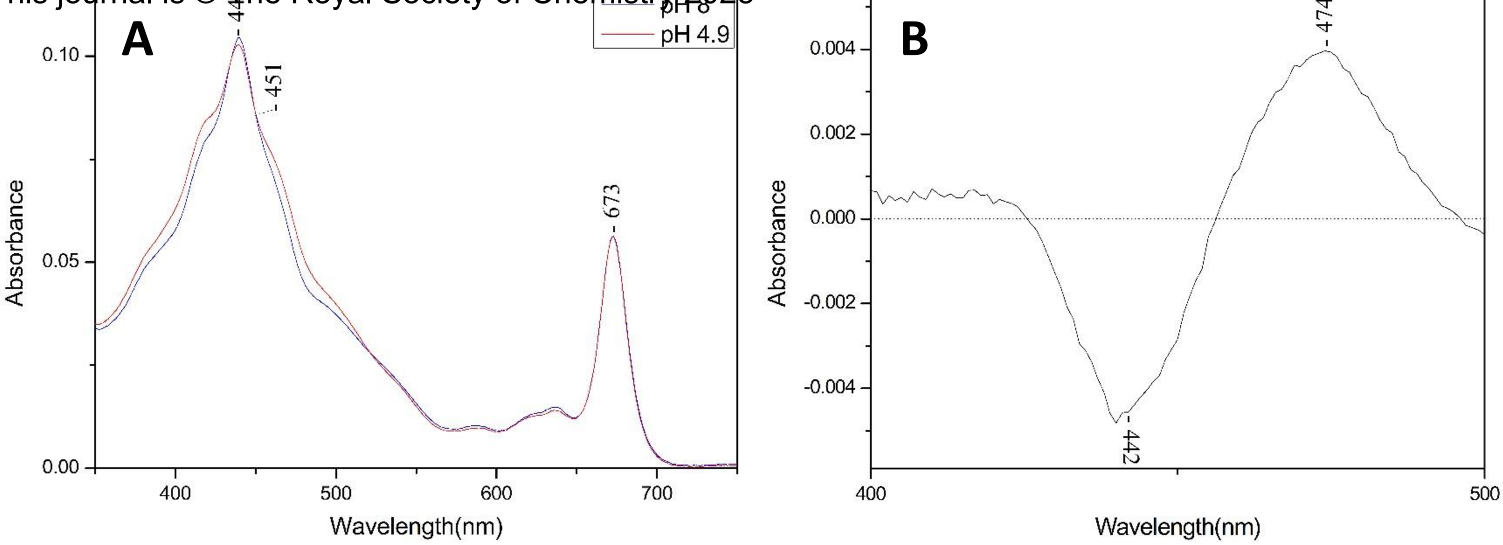

Supplemental Figure S1: A) Absorption spectra of at room temperature of FCPs of Fragilariopsis sp without normalization. pH8 (blue line) and pH4.9 (red line). Initial baseline position was fixed at zero. B) Difference absorption spectra obtained by subtracting the spectra measured at pH 8 from pH 4.9. No corrections were made.

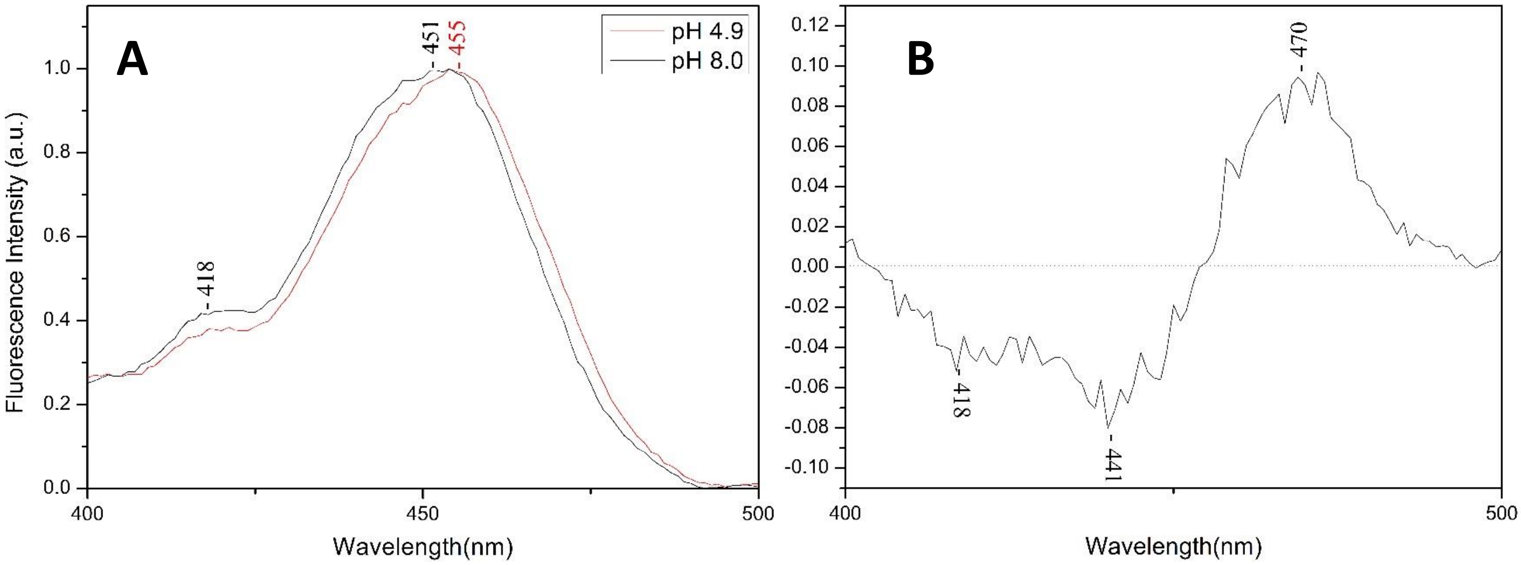

Supplemental Figure S2: A) Fluorescence excitation spectra of Fragilariopsis sp from the spontaneous emission from Chl-c1/c2 at 640 nm at pH 8 (black line) and pH 4.9 (red line) normalized at maxima. B) Difference fluorescence spectra obtained by subtracting the spectra measured at pH 8 from pH 4.9. No corrections were made.

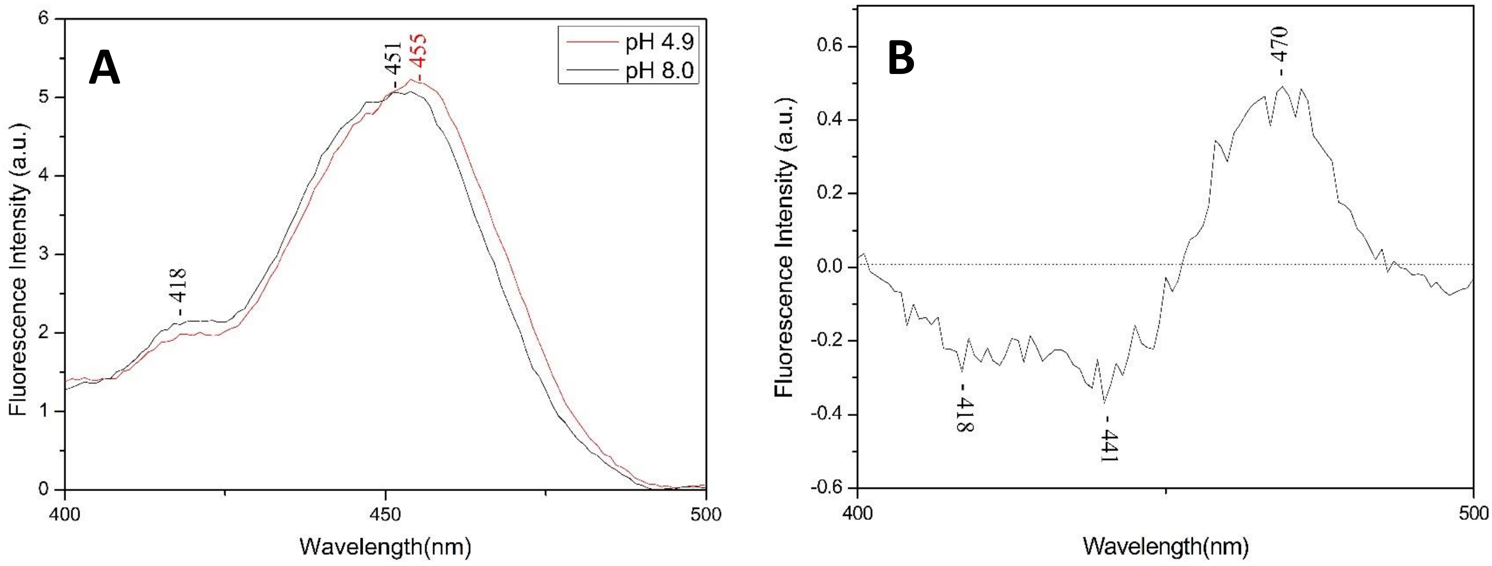

Supplemental Figure S3: A) Fluorescence excitation spectra of Fragilariopsis sp from the spontaneous emission from Chl-c1/c2 at 640 nm at pH 8 (black line) and pH 4.9 (red line) without normalization. B) Difference fluorescence spectra obtained by subtracting the spectra measured at pH 8 from pH 4.9. No corrections were made.
